# Leiomyoma of the breast parenchyma: a case report and review of the literature

**DOI:** 10.1590/1516-3180.2016.0253040117

**Published:** 2017-06-15

**Authors:** Rodrigo Gregório Brandão, Simone Elias, Afonso Celso Pinto Nazário, Maria do Carmo Guedes Alcoforado Assunção, Camilla Cirone Esposito Papa, Gil Facina

**Affiliations:** I Medical Doctor and Doctoral Student, Discipline of Mastology, Department of Gynecology, Universidade Federal de São Paulo (UNIFESP), São Paulo (SP), Brazil.; II Medical Doctor, Doctorate in Medicine and Assistant Professor, Discipline of Mastology, Department of Gynecology, Universidade Federal de São Paulo (UNIFESP), São Paulo (SP), Brazil.; III Medical Doctor, Doctorate in Medicine and Full Professor of the Department of Gynecology, Universidade Federal de São Paulo (UNIFESP), São Paulo (SP), Brazil.; IV Medical Doctor, Doctorate in Medicine and Head of the Locus Laboratory, Department of Pathology, Universidade de São Paulo (USP), São Paulo (SP), Brazil.; V Undergraduate Student, Faculdade Santa Marcelina (FASM), São Paulo (SP), Brazil.; VI Medical Doctor, Doctorate in Medicine, Full Professor and Head of the Discipline of Mastology, Department of Gynecology, Universidade Federal de São Paulo (UNIFESP), São Paulo (SP), Brazil.

**Keywords:** Breast, Ultrasonography, Leiomyoma, Breast neoplasms, Diagnosis

## Abstract

**INTRODUCTION:**

Benign tumors are often seen in breast screening examinations. However, the differential diagnosis is not always simple because of radiological similarity between the different benign lesions.

**CASE REPORT:**

We present a rare case report of leiomyoma of the breast parenchyma in a 68-year-old asymptomatic patient. The mammographic and ultrasonographic findings were similar to those observed in benign lesions.

**CONCLUSION:**

The histopathological diagnosis requires careful differentiation from lesions that have smooth muscle proliferation, especially leiomyosarcoma. The most commonly performed treatment is resection of the lesion with free margins. Although breast leiomyoma is rare, it should be considered among the differential diagnoses for breast nodules of benign appearance. Resection with safety margins proved to be the only treatment needed.

## INTRODUCTION

Leiomyoma is considered to be the rarest non-epithelial tumor of the breast.^1^ It occurs more frequently in the retroareolar region because of the greater amount of smooth muscle in this location.^2^ Its presence in the mammary parenchyma is extremely rare, with fewer than 30 cases reported so far in the literature.^3^ The clinical, radiological and pathological characteristics do not differ markedly from those observed in the most frequent benign lesions. We report a case of leiomyoma in the breast parenchyma that was seen in our service and conducted a review of the literature, with special attention to radiological features that have been described so far.

## CASE REPORT

A 68-year-old woman was seen at the Division of Mastology, Department of Gynecology of the Federal University of São Paulo (Universidade Federal de São Paulo, UNIFESP) with a non-palpable tumor that had been detected through screening mammography. The patient presented controlled hypertension and minor degenerative osteoarticular alterations. She reported having had three pregnancies and two deliveries, with thirty months of breastfeeding. She said that she did not have any other symptoms such as papillary flow or cutaneous lesions. She reported having had routine annual mammograms and that she had not had any previous surgery or biopsies. The mammogram performed two years earlier did not show any abnormalities. The physical examination was unremarkable, with no evidence of any palpable mass, skin changes or axillary lymphadenopathy.

### Imaging findings

**Figure 1.**
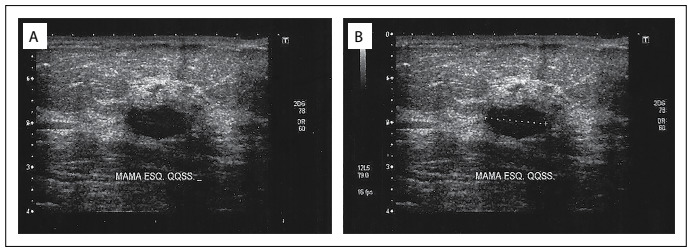
Sonographic findings in breast leiomyoma, demonstrating a hypoechoic oval mass that was predominantly circumscribed but sometimes showed microlobulated margins, and which was parallel to the breast skin. It was classified via ultrasonography in Breast Imaging-Reporting and Data System (BI-RADS) category 4.

Mammographic images showed an isodense circumscribed oval mass measuring 1.8 x 1.0 cm that was located at the junction of the upper quadrants of the left breast. Sonographic images of the left breast showed a hypoechoic homogenous oval mass measuring 1.4 x 0.7 cm that was horizontal and parallel to the skin. It had two lobulations and circumscribed margins, and was coincident with the location described through mammography (Figure 1). The lesion did not present any posterior acoustic shadow, hyperechoic halo or other associated abnormal features. The mass was classified as being in Breast Imaging-Reporting and Data System (BI-RADS) category 4.

### Histopathological findings

**Figure 2.**
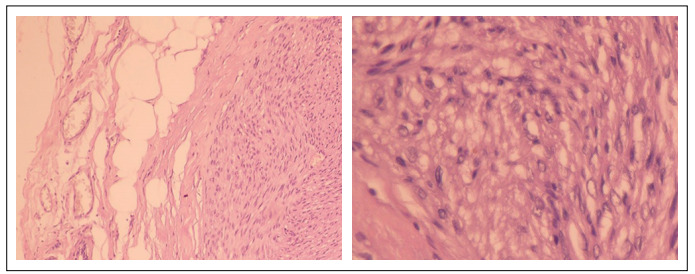
Histological sections revealing circumscribed appearance of the lesion, with proliferation of fusiform pattern and lack of atypical forms. Staining with hematoxylin and eosin (10 x and 40 x).

An ultrasound-guided breast core biopsy with a 12-gauge needle was performed and five fragments were obtained. The pathological evaluation showed a mesenchymal neoplasm with muscle differentiation. The patient underwent surgical excision of the lesion. The histological findings revealed a circumscribed lesion with a pattern of fusiform proliferation and formation of interlaced bundles and fascicles (Figure 2). No cellular atypia, necrosis or mitotic figures was found. Immunohistochemical stains for CD34 and S100 were positive, and negative for desmin and smooth muscle actin. A diagnosis of smooth muscle tumor, and specifically mammary parenchyma leiomyoma, was established. There was no recurrence of the lesion after follow-up of 60 months.

## DISCUSSION

Even though Strong’s first paper on breast leiomyoma was published in 1913, knowledge of the etiology of this condition remains uncertain.^4^ It has been taken to originate from smooth-muscle angiomatous cells, given the “angiocentric” proliferation of smooth muscle that is observed. This theory is reinforced through the observation that blood vessels are present at locations showing defects or artefacts of histological fixation.^5^ Current immunohistochemical findings rule out teratogenic origins. Uncertainty remains regarding theories of embryological displacement of smooth muscle cells of the areola, and regarding an origin from multipotent mesenchymal cells. These were proposed in the first half of the twentieth century by Melnick^6^ and Shauder.^7^

Breast leiomyoma occurs predominantly in women, with only one case reported in a man.^2^ The age of highest incidence is between 40 and 60 years. It presents as an isolated tumor of slow growth, with similar characteristics to the most common benign tumors.^8^^,^^9^^,^^10^ The presence of pain was observed in only three cases, being more frequent in tumors of areolar location due to the contraction of neoplastic muscle cells.^11^

Physical examination usually reveals a mobile nodule with well-defined limits and fibroelastic consistency, although sometimes it has been reported to have hardened consistency.^12^^,^^13^^,^^14^ Mammographic images have been described as showing an isodense or hyperdense oval mass, with outlines that are most often circumscribed (Table 1).^1^^,^^13^^,^^15^^,^^16^^,^^17^^,^^18^^,^^19^^,^^20^^,^^21^ Microcalcifications relating to leiomyoma have never been described.^15^^,^^16^^,^^22^^,^^23^

**Table 1.** Radiological findings from 10 cases of mammary leiomyoma.

Author	Year	Radiological findings
Lauwers et al.^20^	1990	MMG: Well-defined focal asymmetry; benign appearance without calcifications or loss of contours
		US: None
Nazário et al.^13^	1995	MMG: Hyperdense, homogeneous image with defined regular margins.
		US: None
Kaufman and Hirsch^1^	1996	MMG: Dense breast without identifiable abnormalities.
		US: None
Son et al.^18^	1998	MMG: Oval nodule with well-defined margins
		US: Isoechoic oval nodule; slightly lobulated with well-defined margins
Sidoni et al.^17^	1999	MMG: Bulky oval mass, with circumscribed margins.
		US: Hypoechoic oval mass, with well-defined margins.
Pourbagher et al.^15^	2005	MMG: Nodule with well-defined margins without calcifications.
		US: Circumscribed hypoechoic solid oval mass, with well-defined margins.
Ende et al.^19^	2007	MMG: Isodense oval nodule with indistinct margins.
		US: Not viewed.
Minami et al.^16^	2011	MMG: Hyperdense oval nodule with indistinct margins, without spicules or microcalcifications.
		US: Hypoechoic nodule, with well-defined margins.
Shah et al.^30^	2013	MMG: Circumscribed isodense oval nodule.
		US: None
Brandão et al.(present case)	2015	MMG: Circumscribed isodense oval nodule.
		US: Circumscribed hypoechoic oval nodule with rare lobulations.

The effectiveness of mammography is limited in relation to lesions measuring less than 1.0 cm and breasts with predominant glands. Sonography frequently shows a hypoechoic mass with well-defined limits and oval shape.^17^ Presence of lobulations has frequently been observed. Growth parallel to the skin has been observed in 100% of the cases. No well-defined posterior acoustic shadowing has been described.^18^

Magnetic resonance imaging findings were first reported by Minami et al. They described a circumscribed oval nodule, with hypersignal in T1 and T2, and homogeneous enhancement after gadolinium infusion. They pointed out that presence of degeneration can influence the signal pattern in different sequences, as noted in leiomyoma in other regions of the body.^16^

The differential diagnosis should be done in relation to lesions that have smooth muscle proliferation in the absence of epithelial or ductal structures. In this context, the lesions that comprise the differential diagnosis are angioleiomyoma, fibroadenoma and malignant phyllodes tumor.^24^^,^^25^^,^^26^ Because mature adipose tissue is needed to identify cases of hamartoma, this lesion does not provide difficulties in the differential diagnosis.^5^ In cases of lesions suggestive of leiomyoma, leiomyosarcoma is the main situation that needs to be ruled out.^19^^,^^27^

Presence of 2-16 mitotic figures per 10 high-power figures is the main feature for diagnosing leiomyosarcoma. According to Pourbagher, presence of 1-3 mitotic figures might be considered to represent an intermediate category because of the higher risk of local recurrence and, therefore, treatment that is more radical.^15^ Boscaino et al. reported local recurrence in two cases initially diagnosed as leiomyoma. Histological reevaluation of the lesions found presence of increased mitotic activity, and the lesions were reclassified as smooth-muscle neoplasms of undetermined prognosis.^28^ In patients with a confirmed diagnosis of breast leiomyoma, no cases of local recurrence have been reported to date.^11^^,^^12^^,^^13^^,^^14^^,^^15^^,^^16^^,^^19^^,^^22^^,^^23^^,^^24^^,^^25^^,^^26^^,^^27^

We reviewed the literature in MEDLINE and Lilacs using the English keywords “leiomyoma”, “fibroid tumors”, “benign tumor”, “benign neoplasms”, “breast tumor”, “breast neoplasms” and “ultrasonography”. We found 30 case reports that described patients with leiomyoma in the breast parenchyma (Table 2).

**Table 2.** Search of the literature in medical databases for case reports on leiomyoma in the breast parenchyma. The search was conducted on December 5, 2016

Database	Search strategies	URL	Papers found	Related papers
MEDLINE (via PubMed)	((“leiomyoma”[MeSH Terms] OR “leiomyoma”[All Fields]) OR “fibroid tumors”[All Fields] OR (“leiomyoma”[MeSH Terms] OR “leiomyoma”[All Fields] OR “fibromyoma”[All Fields]) OR “benign tumor”[All Fields] OR “benign neoplasms”[All Fields] OR “benign tumor”[All Fields]) AND (“breast/pathology”[Mesh Terms] OR “breast neoplasms”[MeSH Terms] OR “breast cancer”[All Fields] OR “breast tumor”[All Fields] OR “mammary cancer”[All Fields] OR “cancer of breast”[All Fields]) AND (“ultrasonography”[MeSH Terms] AND (“diagnostic imaging”[Subheading] OR (“diagnostic”[All Fields] AND “imaging”[All Fields]) OR “diagnostic imaging”[All Fields] OR “ultrasonography”[All Fields] OR “ultrasonography”[MeSH Terms]) OR (“diagnostic imaging”[Subheading] OR (“diagnostic”[All Fields] AND “imaging”[All Fields]) OR “diagnostic imaging”[All Fields] OR “ultrasound”[All Fields] OR “ultrasonography”[MeSH Terms] OR “ultrasonography”[All Fields] OR “ultrasound”[All Fields] OR “ultrasonics”[MeSH Terms] OR “ultrasonics”[All Fields]) OR (“ultrasonography, mammary”[MeSH Terms] OR (“ultrasonography”[All Fields] AND “mammary”[All Fields]) OR “mammary ultrasonography”[All Fields] OR (“mammary”[All Fields] AND “ultrasonography”[All Fields])))	http://bit.ly/2mCc4uj	117	24
LILACS (via Bireme)	mh: leiomyoma OR tw:leiomyoma OR tw:“fibroid tumors” OR tw:fibromyoma OR tw:“benign tumor” OR tw:“benign neoplasms” OR tw:“benign tumor”)) AND (tw:((“breast/pa” OR mh:c04.588.180 OR mh:c17.800.090.500 OR mh:“breast neoplasms” OR tw:“breast cancer” OR tw:“mammary cancer” OR tw:“breast neoplasms”))) AND (tw:ultrasound OR tw:ultrasonography)) AND (instance:“regional”) AND (db:(“LILACS”))) AND (instance:“regional”) AND ( mj:(“Mama”))	https://goo.gl/HbOEvW	42	6

In reviewing treatments that have been implemented, a wide range of interventions can be identified, from lumpectomy to radical mastectomy (Table 3).^1^^,^^2^^,^^4^^,^^5^^,^^6^^,^^7^^,^^8^^,^^9^^,^^10^^,^^13^^,^^15^^,^^16^^,^^17^^,^^18^^,^^19^^,^^20^^,^^21^^,^^24^^,^^29^ However, since the report by Lauwers in 1990, the standard treatment has been resection with free margins.^20^^,^^21^

**Table 3.** Clinical findings from 20 cases of breast leiomyoma

Author	Year	Sex	Race	Age (years)	Symptoms	Location	Size (cm)	Therapy
Strong^4^	1913	F	W	46	Discomfort	UOQ R	6.0	-
Schauder^7^	1927	F	W	34	Discomfort	UOQ R	3.0	Nodulectomy
Melnick^6^	1932	F	W	45	Pain	JLI R	“small”	Total mastectomy
Leibowich and Lenz^8^	1940	F	W	58	Discomfort	Midline	13.8	Total mastectomy
Stein^9^	1943	F	W	54	Discomfort	UIQ R	4.0	Radical mastectomy
Craig^10^	1947	F	B	40	Pain	LOQ L	10	Nodulectomy
Libcke^24^	1969	F	W	50	Hardening	JUQ R	0.5	Nodulectomy
Haagensen^29^	1971	F	-	52	None	Midline	2.5	Nodulectomy
Diaz-Arias et al.^5^	1989	F	W	69	None	UOQ R	2.0	Nodulectomy
Lauwers et al.^20^	1990	F	B	43	Mammographic finding	JUQ L	0.5	Resection with free margins
Nazario et al.^13^	1995	F	B	53	Nodule	UIQ L	10	Resection with free margins
Kaufman and Hirsch^1^	1996	F	W	48	Nodule	Midline R	1.0	Resection with free margins
Son et al.^18^	1998	F	A	50	Pain	UOQ R	1.0	Resection with free margins
Sidoni et al.^17^	1999	F	-	48	Nodule	UOQ L	4.0	Resection with free margins
Pourbagher et al. ^15^	2005	F	-	47	Mammographic finding	JIQ L	2.5	Resection with free margins
Ende et al.^19^	2007	F	-	48	Mammographic finding	JIQ L	1.2	Resection with free margins
Minami et al.^16^	2011	F	A	63	Mammographic finding	UIQ R	1.6	Excisional biopsy
Shah et al.^21^	2013	F	W	27	Nodule	UIQ L	2.0	Excisional biopsy
Strader et al.^2^	2013	M	W	70	Nodule	JOQ L	7.0	Resection with free margins
Brandão et al. (current case)	2015	F	W	68	Mammographic finding	JUQ L	1.4	Resection with free margins

## CONCLUSION

In conclusion, it can be said that leiomyoma in mammary tissue is an extremely rare condition. The clinical presentation does not differ from that observed in the most common benign tumors of the breast. The radiological findings are characteristically benign, which helps rule out the hypothesis of cancer. In histopathological evaluations, it is important to pay attention to the differential diagnosis of leiomyosarcoma. The standard recommended treatment is local resection with free margins. In this situation, the risk of local recurrence is practically zero.
